# Preparation of Permanent Magnetic Resin Crosslinking by Diallyl Itaconate and Its Adsorptive and Anti-fouling Behaviors for Humic Acid Removal

**DOI:** 10.1038/s41598-017-17360-8

**Published:** 2017-12-06

**Authors:** Qimeng Li, Ji Wu, Ming Hua, Guang Zhang, Wentao Li, Chendong Shuang, Aimin Li

**Affiliations:** State Key Laboratory of Pollution Control and Resources Reuse, School of the Environment, Nanjing University, Nanjing, 210023 P. R. China

## Abstract

In this research, a series of permanent magnetic anion exchange resins (MAERs) were prepared by polymerizing glycidyl methacrylate monomer and crosslinking diallyl itaconate (DAI) and divinylbenzene. The properties and performances of these novel MAERs were systematically characterized and evaluated for humic acid (HA) adsorption by batch experiments. With the increase of DAI content from 0 to 15%, the moisture of MAERs was elevated from 50.23% to 68.53%, along with the adsorption capacity increasing from 2.57 to 3.14 mmol g^−1^. As the concentrations of co-existing cation (Ca^2+^ and Mg^2+^) increased, the adsorption amounts of HA dropped drastically at first and increased a little at high cation concentrations. Although ion exchange was the primary mechanism for HA adsorption, other physical interactions and electrostatic attraction between HA molecules and newly formed oxonium group also played significant roles for HA adsorption. The MAERs could be efficiently regenerated by a mixture of NaCl/NaOH solution (10%/1%), and notably, the MAER-3 with the highest DAI content displayed unapparent loss of adsorption capacity during twenty-one successive adsorption-desorption cycles. These results suggested a novel resin adsorbent for its excellent performances on adsorption, regeneration, and sedimentation in water treatment for natural organic matter removal.

## Introduction

Natural organic matter (NOM), commonly present in various water systems, can cause serious environmental problems, such as undesirable color and formation of disinfection byproducts during the chlorination disinfection, thus are potentially harmful to human health^1,2^. Therefore, it is of crucial importance to reduce NOM concentration in drinking water and other processing water streams.

Many groups have researched on numerous methods for NOM removal, including biological degradation^3^, enhanced coagulation^4^, oxidation^5^, adsorption^6^, membrane filtration^7^, and ion exchange^8,9^. Among them, ion-exchange has attracted extensive attention to remove various pollutants from wastewater for its high regeneration efficiency and simple operation^10,11^. MIEX resin, developed by Orica Co. Ltd, is currently the most widely used magnetic resin for NOM removal^12^. Multiple water treatment projects based on MIEX resin for water purification have been established worldwide, which are mainly owing to its recognized advantages: macroporous structure, high exchange capacity and improved kinetics^13–15^. Furthermore, due to its high density and excellent settling property, MIEX resin could be applied in the form of fluidized bed reactor in a continuous process with partial resin regeneration, which greatly improves the treatment flux^14^.

Although magnetic resins have been found appealing in a large number of industrial-scale water treatment applications and installed new plants continuously, the magnetic resins still attract less attention than they deserve. There is a strong need for developing more efficient and commercially available magnetic resins on the aspects of adsorption, desorption and settlement. The primary concern is how the magnetic iron oxide (γ-Fe_2_O_3_) is embedded in the resin matrix^15^. The resulting agglomeration and rapid settling of dense magnetic microbeads in solution are the sticking points for magnetic resin adsorption processes^15,16^. On the other hand, it is surprising that fewer research papers focus on the resin fouling by NOM. Although the decline in NOM removal can be ignored in some cases during several regeneration cycles, resin adsorption performance can be significantly influenced because hundreds to thousands of adsorption-regeneration cycles occur in real applications^17–19^. Generally, the optimization of resin adsorbents for improving their anti-fouling performance depends on improving their polymer matrix and porosity^18,20,21^. Polyacrylic matrix and macroporous structure are reported to be favorable for resin regeneration because of their more hydrophilic and open structure^15^. Shuang *et al*.^14^ recently developed a novel magnetic anion exchange resin called NDMP, which incorporated with different amount of nanoscale Fe_3_O_4_ and exhibited significantly improved adsorption and reusability performances due to its much more enhanced resin hydrophilicity. The use of polar and hydrophilic crosslinker can also improve the hydrophilicity and change the structure of the resin matrix. However, few studies are reported in this field.

Given that humic substances are flexible and contain plenty of carboxylic, phenolic, and carbonyl groups, they can be easily adsorbed by anion exchange resins and, on the other hand, cause severe resin fouling problem^14,20^. The mutual interactions between resin and fouling pollutants were found to be diversiform in previous studies. For example, Fu *et al*.^22^ concluded that NOM was removed via ion exchange, yet others have shown evidence of both ion exchange and adsorption^23^. Therefore, the influences of resin properties and adsorption mechanisms should be further studied.

In this work, a series of novel permanent-magnetic anion exchange resins (MAERs) with the different amount of crosslinker DAI in feed compositions were prepared. A commercial Alfa humic acid (HA) was chosen as the model adsorbate. The purposes of this work were to (1) systematically study the adsorption behaviors of HA by MAERs under different conditions; (2) investigate the regeneration and reusability performance of MAERs on HA removal; and (3) elucidate the adsorption mechanisms between HA and MAERs.

## Experimental Section

### Materials

Glycidyl methacrylate (GMA, >99%), divinylbenzene (DVB, 63.3%), benzoyl peroxide (BPO), trimethylamine hydrochloride, cyclohexanol and polyvinyl alcohol (PVA, GH20) were all industrial products and supplied by J&K Chemical Co. Ltd., China. Commercial γ-Fe_2_O_3_ was obtained from Tangyin Zhongke magnetoelectric Co., Ltd. Tetraethoxysilane (TEOS) and dimethyldiethoxylsilane (DMDES) were purchased from Nanjing Capture Chemical Co., Ltd. All these reagents were used as supplied without further purification. Diallyl itaconate (DAI) was obtained by the esterification reaction of propanol and itaconic acid. Detailed preparation procedures were described in Text S1, supplementary information.

### Synthesis of MAERs

As the γ-Fe_2_O_3_ particles used in this work were commercial micrometer-scale products and had been calcined for surface passivation, the big spatial hindrance and poor surface active groups made the γ-Fe_2_O_3_ particles much more difficult for surface modification. However, by modification with silane coupling agents (TEOS + DMDES) for two times, the γ-Fe_2_O_3_ could be successfully encapsulated into the resin matrix, which was similar to Wang’s method^24^.

Three magnetic resins noted as MAER-1 to MAER-3 were synthesized through suspension polymerization and amination reaction. For polyacrylic resins, the DAI could readily polymerize with the monomers due to their high reactivity ratio and similar compatibility (logD 1.70, logP 1.704 ± 0.439). With the different amount of crosslinker DAI and DVB in feed, the resins with different hydrophilicity and polarity could be prepared. Detailed synthetic steps and compositions of oil phase for each resin were illustrated in Text S2 and Table S1.

### Characterization of MAERs

The functional groups of resins were identified by FTIR spectroscopy (Nexus870, Nicolet). The morphologies of microbeads were characterized via scanning electron microscopy (SEM, S-3400, Hitachi). The specific surface area and pore distribution of the resins were measured by N_2_ adsorption–desorption experiments at 77 K (ASAP, Micromeritics, USA) based on the standard Brunauer–Emmett–Teller (BET) method. The size distributions of magnetic precursor resins were determined by a laser diffraction particle size analyzer (Mastersizer 3000, England). The resins before and after the adsorption of HA were characterized with X-ray Photoelectron Spectroscopy (XPS, PHI 5000 VersaProbe, UlVAC-PHI Japan). All XPS spectra were calibrated against C1s bands at 284.6 eV.

### Batch adsorption

Batch adsorption experiments were performed to investigate the HA adsorption behaviors of MAERs. For adsorption kinetics, 0.500 g of dried resin with 500 mL HA solution of known concentration was stirred in a 1000 mL conical flask at 293 K. Water samples were withdrawn at different time intervals, and determined by a total organic carbon analyzer (OIA1088, USA).

The adsorption isotherms were performed at the temperatures of 278, 293, and 308 K. 0.100 g of MAERs and 100 mL HA solution of known concentrations were shaken for 48 h to attain equilibrium. Afterwards, the residual HA concentrations were determined.

The amount of adsorbed HA *Q*_*t*_ (mg g^−1^) was estimated as:

1 $${Q}_{t}=V({C}_{0}-{C}_{t})/W$$

where, *C*_0_ and *C*_*t*_ (mg L^−1^) are concentrations of adsorbate at the beginning and time *t*, respectively. *V* (*L*) is the solution volume. *W* (g) is the weight of dried resin. The variables *Q*_*t*_ and *C*_*t*_ can be replaced by *Q*_*e*_ and *C*_*e*_ at the equilibrium state, respectively.

For the pH-effect experiments, the initial pH was adjusted in the pH range of 3.0 to 11.0 using 1.0 N HCl or NaOH solution. Adsorption experiments were performed by shaking a series of conical flasks containing 0.100 g resin and 100 mL HA solutions (50 mg L^−1^) at different pH values and 293 K for 48 h. At each pH value, the adsorption experiments were repeated for three times to guarantee the replicability of results. To determine the effects of alkali-earth metal ions on HA adsorption, the HA solutions (50 mg L^−1^) with different concentrations of Ca^2+^ and Mg^2+^ were shaken with 0.100 g MAERs to investigate their effects on HA adsorption.

### Desorption and reusability

The desorption and regeneration behaviors of MAERs could directly reflect their anti-fouling performance. Initially, batch adsorption experiments were conducted at 293 K until equilibrium. Next, the saturated resins were regenerated by NaCl solution (10%, w/w) or a mixture of NaCl/NaOH solution (10%/1%, w/w) for different time intervals. The desorption efficiency (*DE*, %) was calculated by the following Eq. (2):

2 $$DE=100 \% \cdot {V}_{d}{C}_{d}/W{q}_{e}$$

where *V*_*d*_ (L) is desorption agent volume, and *C*_*d*_ (mg L^−1^) is the final concentration of HA solution. The adsorption-desorption processes were undertaken for 21 times to verify the loss of adsorption capacity, and alternatively to know the extent of regeneration.

Continuous HA adsorption jar tests were carried out with 1.0 mL resin and 100 mL HA solution of 50 mg L^−1^ at 293 K and 150 rpm. After adsorption for 1 h, 1 mL of supernatant was withdrawn and analyzed. Then, the resins were separated by a magnet and transferred into another 100 mL untreated solution, which was similar as the former operation run.

## Results and Discussion

### Characterization

The essential physicochemical characteristics of MAERs were shown in Table 1. The BET surface area and total pore volume of MAERs were in the range of 1.1–1.8 m^2^ g^−1^ and 6.5–7.5 mm^3^ g^−1^, respectively. For the three resins, the moisture of resins increased from 50.23% to 68.53%, with the DAI content in composition increasing from 0 to 15%. Besides, due to the superior crosslinking capability of DAI, the resin with higher DAI content had a lower average pore size and stronger mechanical strength (indicated by the sphericity rate of precursor resins after osmotic-attrition). Unexpectedly, the anion exchange capacity (AEC) of MAERs was also affected by the DAI content, which is expressed by the content of N element for each resin. Therefore, the improved hydrophilicity as a function of DAI content possibly allowed the functionalizing agent (trimethylamine hydrochloride) to penetrate deeply into the resin matrix, leading to an increased AEC (tested by the content of quaternary ammonium groups).

**Table 1:** Physicochemical properties of MAERs.

Resin	Specific surface area (m^2^/g)	External surface area (m^2^/g)	Pore volume (mm^3^/g)	Average pore diameter (nm)	Water content (%)	N (%)	AEC^1^ (mmol/g)	Sphericity^2^ (%)
MAER-1	1.109	0.768	7.311	26.367	50.23	2.77	2.57	72.23
MAER-2	1.396	0.739	6.527	18.704	62.14	3.05	2.93	80.27
MAER-3	1.785	1.490	7.518	16.845	68.53	3.45	3.14	86.05

^1^Anion exchange capacity.

^2^Sphericity after osmotic-attrition (%) was determined with the precursor resins.

**Figure 1:**
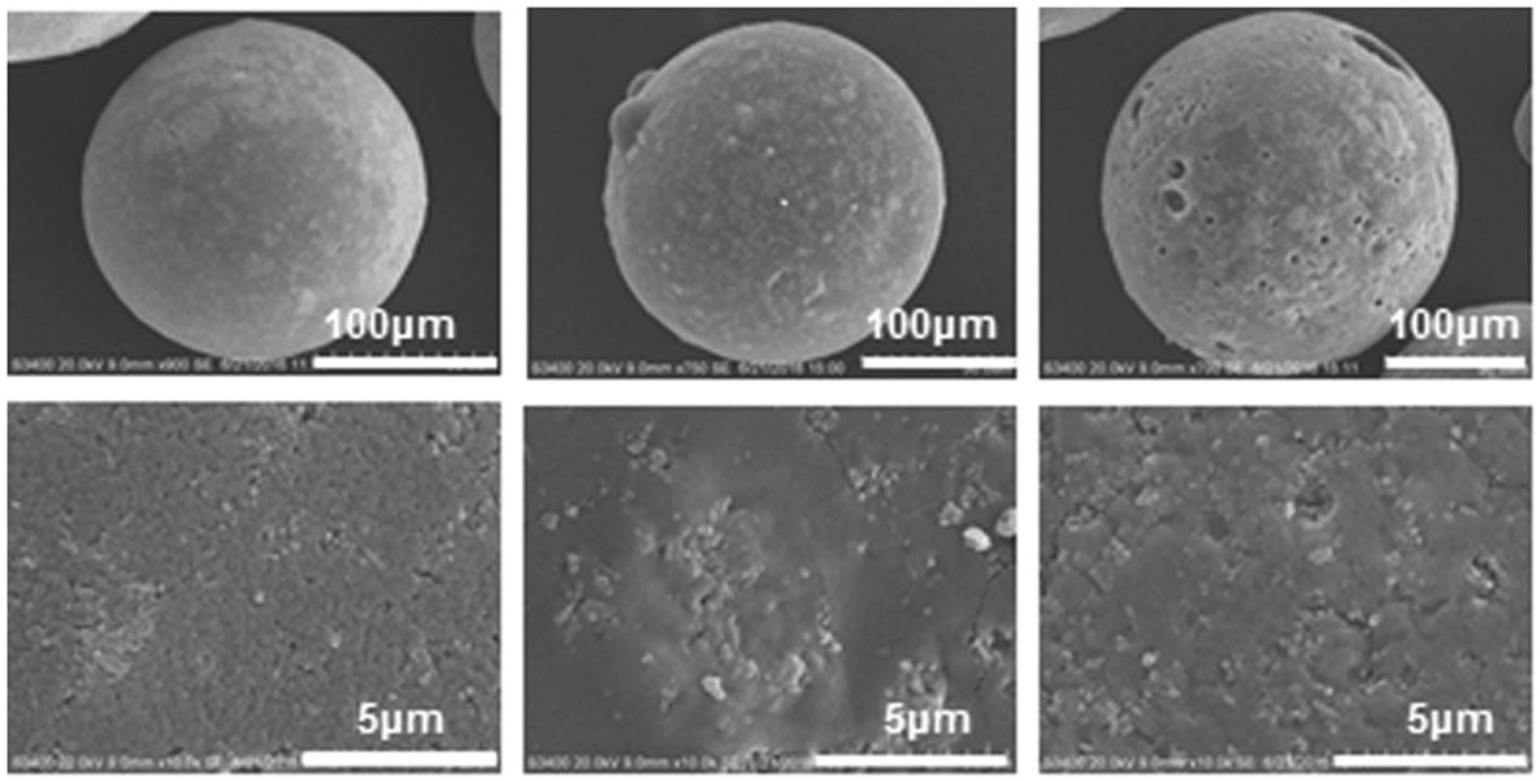
SEM micrographs of (**a**) MAER-1, (**b**) MAER-2, and (**c**) MAER-3.

To observe morphological characteristics of MAERs, the SEM analysis was performed and illustrated in Fig. 1. The size distributions of MAERs are shown in Supplementary Fig. S1. It is clearly seen that the beads are satisfactorily monodisperse in the range of 50–150 μm, much smaller than conventional resins. Particularly, the surface of MAER-3 shows many cavities and canals, which are deemed as essential prerequisites for efficiently adsorbing high-molecular-weight pollutants like HA.

**Figure 2:**
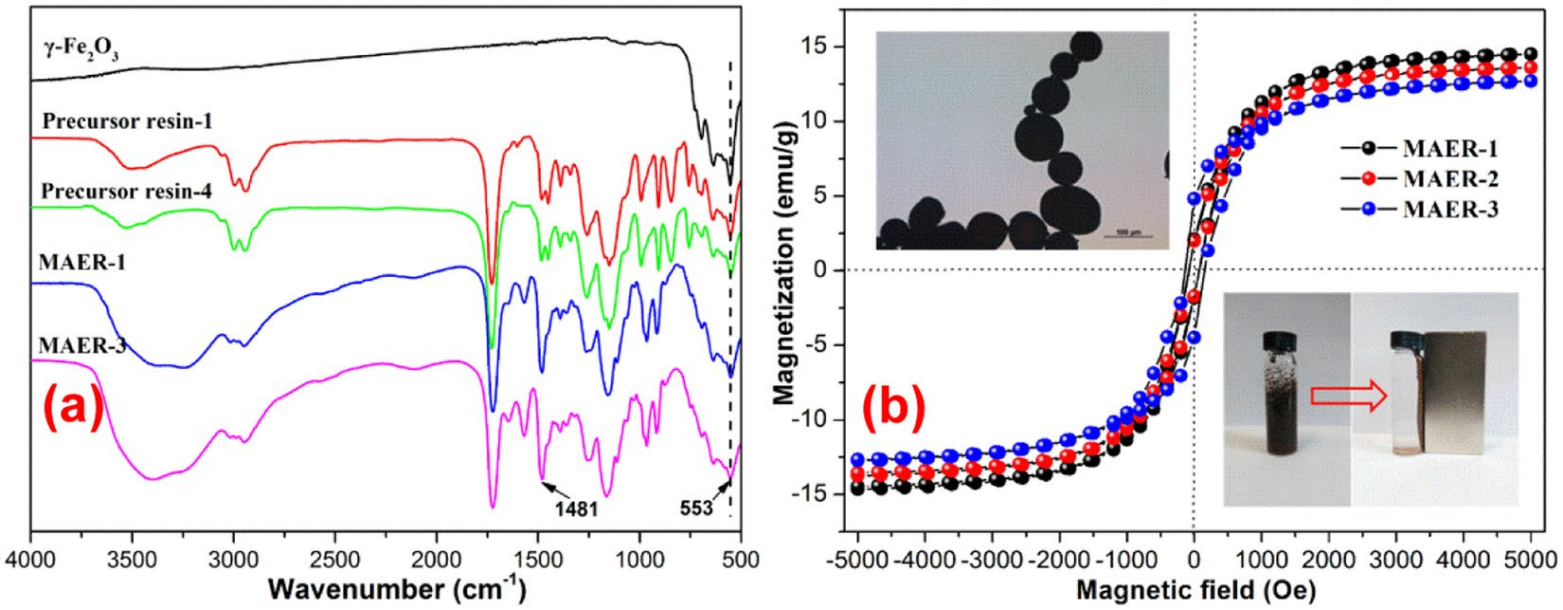
The characterization of MAERs: (**a**) FTIR spectra, and (**b**) magnetic hysteresis loops.

The FTIR spectra of MAERs are presented in Fig. 2a. The absorption bands at 904 and 844 cm^−1^ of precursors are corresponding to the epoxy groups, which disappeared after the amination reaction. The absorption band at 1481 cm^−1^ is attributed to the C–N stretching of quaternary ammonium groups. The broad absorption band ranging from 3200 to 3500 cm^−1^ originates from O–H deformation vibration of hydroxyl groups, proving the success of amination. As shown in Fig. 2b, the saturation magnetizations of resins are in the order of MAER-3 < MAER-2 < MAER-1, and consistent with the increased mass of newly formed functional groups. Furthermore, the curves do not pass through the origin of coordinate, suggesting that the MAERs can behave as a permanent magnet in the absence of an external magnetic field. As a result, the magnetic microbeads could easily agglomerate with others and form flocks without the use of a magnet, which would result in the rapid settling in water and greatly improve the processing wastewater volume, and make it more amenable to automation.

### Adsorption kinetics

Adsorption kinetics study was conducted to evaluate the HA adsorption performance onto MAERs. To elucidate the adsorption behaviors of three resins, the pseudo-first order, pseudo-second order and Weber-Morris intra-particle diffusion models were applied and expressed in Text S3.

**Figure 3:**
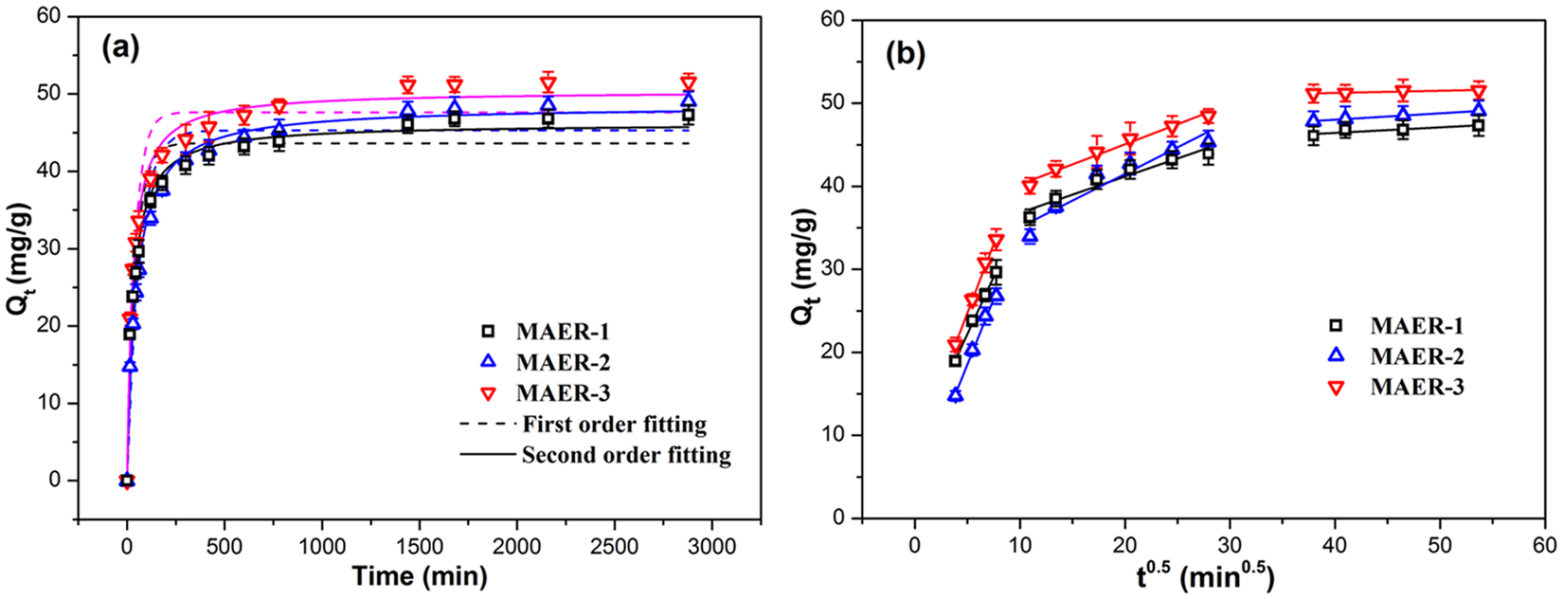
The plots of HA adsorption for (**a**) pseudo first-order and pseudo second-order kinetics model, and (**b**) intra-particle diffusion model (1.0 g resin/L, 100 mg/L of HA solution, 293 K).

As shown in Fig. 3a, the adsorption amounts of HA for all resins increased quickly at the initial stage and then slowed down until equilibrium. The fast adsorption at the initial time was due to the concentration gradient between the HA and amounts of active sites at the beginning of adsorption process. It can be seen in Fig. S2 that a negligible uptake of HA by three precursor resins and γ-Fe_2_O_3_ was observed, demonstrating that the direct adsorption of HA by polymer matrix or γ-Fe_2_O_3_ was excluded. As depicted in Fig. 3b, the adsorption of HA onto MAERs can be well fitted by intra-particle diffusion model (*R* ^2^ > 0.996). The HA adsorption consists of three steps: the movement of adsorbates through the diffusion boundary layer, intra particle diffusion and attainment of equilibrium^25^. The first portions of straight lines do not pass through the origin, indicating that intra-particle diffusion is not the only limiting step, and other steps also influence the rate of mass transfer according to the Weber-Morris theory^14,26^. Obviously, the MAER-3 had the fastest adsorption kinetics, which was in accord with the fact that HA adsorption differs among various surfaces, and hydrophilic surface is much superior to hydrophobic surface if electrostatic attraction mainly acts on adsorption^27^.

The kinetic parameters are listed in Table 2. It is evident that the adsorption process under this condition is more suitable described by the pseudo-second-order kinetic model (*R* ^2^ > 0.986), which is in accordance with their chemisorption process. The kinetic constant *k*_1_ and initial adsorption rate *h* follow the order: MAER-3 > MAER-1 > MAER-2, indicating the resin with higher hydrophilicity and larger pore size has faster kinetics for HA adsorption. Especially, the supermacroporous structure of the surface of MAER-3 lead to the largest diffusion rate constant of the three resins and greatly facilitate the diffusion and adsorption of high-molecular-weight HA.

**Table 2:** Kinetic parameters of three kinetic models at 293 K.

Resin	Pseudo-first-order model			Pseudo-second-order model				Intra-particle diffusion model		
	*k*_1_ */10* ^*−2*^ (min^−1^)	*q*_*e*_ (mg/g)	*R* ^2^	*k*_2_ */10* ^*−4*^ (g/mg min)	*h* (mg/g min)	*q*_*e*_ (mg/g)	*R* ^2^	*K*_*1d*_ (mg(g min)^0.5^)	*I* (mg/g)	*R* ^2^
MAER-1	2.247	43.63	0.9378	7.304	1.558	46.18	0.9883	2.747	8.477	0.9971
MAER-2	1.572	45.28	0.9492	4.582	1.078	48.51	0.9937	3.147	2.816	0.9914
MAER-3	2.429	47.63	0.9302	7.197	1.828	50.40	0.9863	3.313	8.191	0.9965

On the other hand, it was worth mentioning that a direct comparison of HA adsorption capacity between the examined resins with those obtained in the literature is difficult due to the varying experimental conditions. In this study, the adsorption of HA by using other adsorbents were also performed. The physicochemical property of used adsorbents and adsorption capacity were shown in Table S2 and Figure S3, respectively. As can be seen, the D213 resin with the polyacrylic matrix has the largest adsorption capacity of HA, which was about 25% larger than that of MAER-3. This phenomenon was mainly due to the fact that the incorporated γ-Fe_2_O_3_ in MAER-3 could not participate in the HA adsorption. However, other resins with the hydrophobic polystyrene matrix and activated carbon had much lower HA adsorption capacity, indicating that these commercially available adsorbents were not suitable for HA removal.

### Adsorption isotherms

Adsorption isotherm describes the equalibrium between adsorbent in the liquid and adsorbed on the surface of adsorbent at constant temperature. To obtain an enhanced understanding of adsorption mechanism, the isothermal adsorption data are fitted by Langmuir and Freundlich model, respectively. The Langmuir model assumes that adsorption sites are homogeneous and occupied in a one-to-one manner, while Freundlich model is an empirical model based on the assumption of heterogeneous adsorption^13,28^. They are presented as follows:

3 $${\rm{The}}\,{\rm{Langmuir}}\,{\rm{isotherm}}:{Q}_{e}={Q}_{m}{K}_{L}{C}_{e}/(1+{K}_{L}{C}_{e})$$

4 $${\rm{The}}\,{\rm{Freundlich}}\,{\rm{isotherm}}:Qe={K}_{F}C{e}^{1/n}$$

where, *Q*_*e*_ (mg g^−1^) and *C*_*e*_ (mg L^−1^) are the adsorption capacity and concentration at the equilibrium state, respectively. *Q*_*m*_ (mg g^−1^) is the theoretical monolayer saturation capacity. *K*_*L*_ and *K*_*F*_ are the constants of Langmuir and Freundlich model, respectively. *n* is the Freundlich exponent which serves to describe the adsorption intensity^29^.

**Figure 4:**
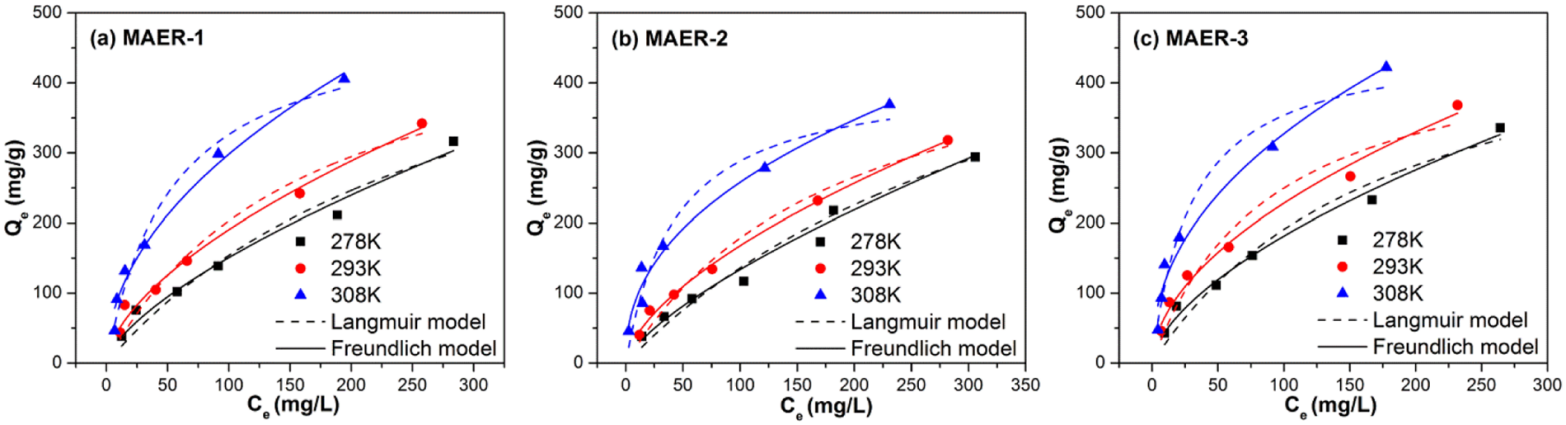
Isotherms of HA adsorption by MAERs at 278, 293 and 308 K.

It can be observed in Fig. 4 that the equilibrium adsorption amounts of HA increased for all resins with increasing temperature, which is consistent with the characteristic of chemical interaction. As illustrated in Table 3, the adsorption data can be better described by Freundlich model with higher values of correlation constant, suggesting other interactions except ion exchange may exist in HA adsorption. Previous researchers have revealed the cation-π bonding and π-π interaction between organic pollutants and various adsorbents^30,31^. More recently, the π-π interaction has been proposed between the adsorbed HA molecules and free HA molecules^14,29^. On the other hand, a high value of *n* implies strong affinity of HA toward the resin. It is clearly seen that the value of *n* increased gradually with the increase of temperature. For all resins, the obtained values of *n* are higher than 1, and accordingly, the adsorption processes are favorable. More importantly, the Freundlich bonding constant and adsorption capacity are generally increased with the increasing content of DAI, indicating the essential roles of DAI for improving the resin adsorption performance.

**Table 3:** Adsorption parameters of Langmuir and Freundlich isotherm models.

Adsorbent	Temperature (K)	Langmuir model			Freundlich model		
		*K*_*L*_ */10* ^*−2*^ (L/mg)	*Q*_*m*_ (mg/g)	*R* ^*2*^	*K*_*F*_ (L/g)	*n*	*R* ^*2*^
MAER-1	278	0.331	502.60	0.9493	6.886	1.492	0.9790
	293	0.610	536.18	0.9550	12.036	1.668	0.9861
	308	1.853	618.72	0.9761	30.586	2.022	0.9799
MAER-2	278	0.254	413.80	0.9738	5.017	1.403	0.9834
	293	0.522	519.97	0.9766	9.797	1.622	0.9955
	308	2.297	670.24	0.9428	35.757	2.330	0.9741
MAER-3	278	0.545	461.80	0.9529	11.194	1.654	0.9875
	293	1.113	474.40	0.9451	19.770	1.882	0.9869
	308	3.278	541.01	0.9577	42.365	2.252	0.9721

To further reflect the feasibility and favorability of HA onto MAERs, the thermodynamic analysis of the adsorption processes was performed. The thermodynamic parameters, including free energy change (*ΔG*), enthalpy change (*ΔH*) and entropy change (*ΔS*), are calculated according to the Eqs (5–7):

5 $$ln(1/Ce)=ln{K}_{0}+(-{\rm{\Delta }}H/RT)$$

6 $${\rm{\Delta }}G=-nRT$$

7 $${\rm{\Delta }}G={\rm{\Delta }}H-T{\rm{\Delta }}S$$

where *T* (K) is the absolute temperature and *n* is the Freundlich constant. *C*_*e*_ is the equilibrium concentration calculated from the well-fitted Freundlich isotherm. *R* is the gas constant (8.314 J mol^−1^ K^−1^).

As can be observed in Table S3, the *ΔG* values are all negative at three temperatures and increased with the increasing temperatures, revealing that HA adsorption by MAERs is spontaneous and thermodynamically favorable. The positive values of *ΔH* prove the adsorption processes are endothermic and mainly dominated by chemical adsorption (40–140 kJ mol^−1^). However, the relatively lower values of *ΔH* than the usual chemical adsorption imply the existence of other physical forces. Furthermore, the positive values of *ΔS* indicate that the HA adsorption is dominated by entropic change. These thermodynamic parameters of HA adsorption are consistent with other studies^10,14,28^.

### Effect of pH

**Figure 5:**
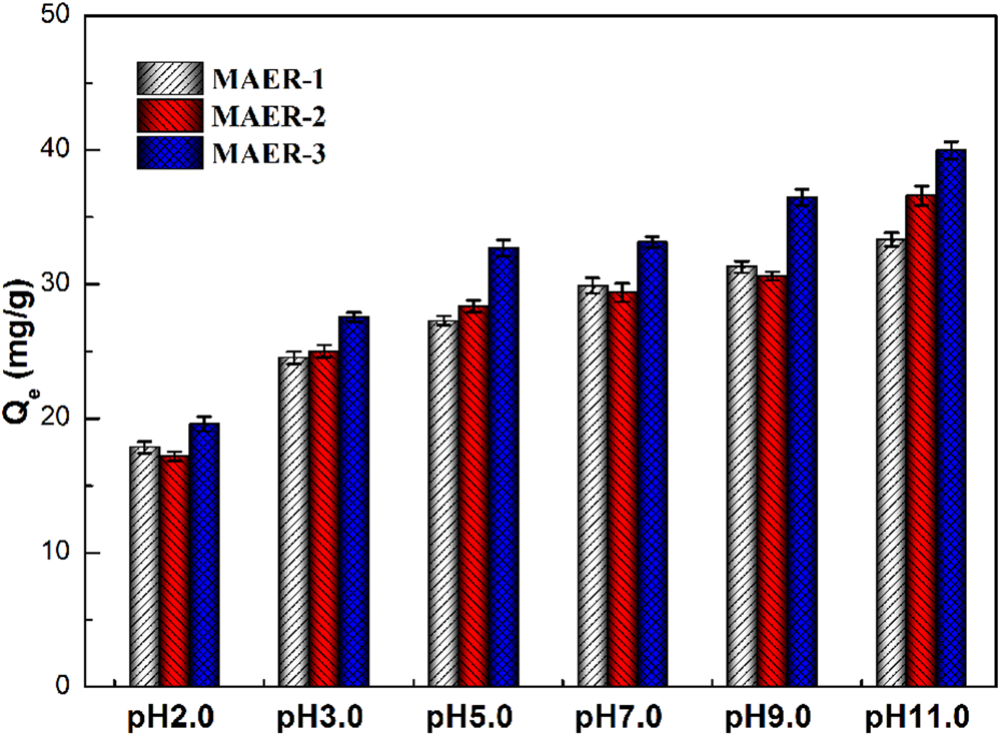
The adsorption amounts of HA onto MAERs at different pH values (1.0 g resin/L, 50 mg/L of HA solution, 293 K).

The solution pH is a key factor which can significantly affect the ionization degree of HA molecules^32^. In this research, the influences of solution pH on HA adsorption were investigated over the pH range of 3.0–11.0. Figure 5 showed that the adsorption behaviors were all pH-dependent. Generally, with the increase of pH value, the HA adsorption amounts increased as well. At most pHs, the adsorbed HA was in the order of MAER-1 ≈ MAER-2 < MAER-3. At pH 11.0, the removal efficiency of HA by MAER-3 was nearly 80%.

The adsorption behaviors could be explained by the variation of zeta potentials. Due to the quaternary ammonium groups of the resin matrix, the MAERs were positively charged and maintained stable at both acidic and basic conditions^33^. On the other hand, the zeta potentials of HA molecules varied considerably in the tested pH range due to their dissociation of carboxyl groups and phenolic hydroxyl groups (Fig. S4a). Interestingly, the surface charge became less negative at strongly basic conditions. This may be interpreted by the variation of molecular shape and size of HA at different pH values. As shown in Fig. S4b, the sizes of HA molecules at weakly acidic conditions were greatly larger than that at basic conditions, while the size of HA molecules at pH 9.5 was in a wide distribution. However, the size distributions of HA molecules at strongly acidic condition were not given for their precipitation and much higher deviation. Under acidic conditions (like pH 2.0), HA macromolecules were near neutral, thus the adsorption was more favored by other driving forces, such as cation-π bonding between quaternary ammonium groups of MAERs and π-electron-rich HA aromatic structures (act as π-donors), and π-π interaction between free HA and absorbed HA^14,34^. Furthermore, the electrostatic attraction between the newly formed positively charged oxonium groups (derived from the oxygen atom of C–O–C in the resin matrix) and HA molecules should also be mentioned^35,36^. Taken together, the increasing amounts of adsorbed HA were consistent with the disaggregation of coalesced HA molecules and formation of chain-shaped structure, which resulted in a more effective diffusion of HA molecules into the interior part of resin.

### Effects of cations

**Figure 6:**
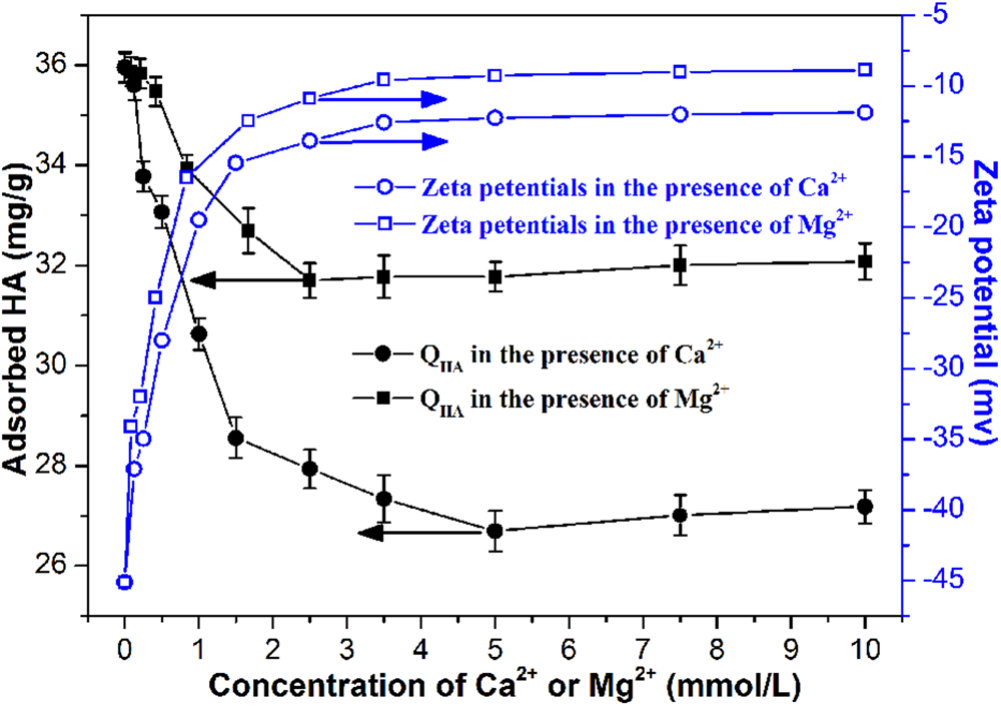
The HA adsorption capacities and zeta potentials as a function of Ca^2+^ and Mg^2+^ concentration (1.0 g resin/L, 50 mg/L of HA solution, 293 K).

As alkaline-earth metal ions always exist in various water systems, it is of vital importance to investigate the impacts of co-existing cations on HA adsorption^28,37^. In our preliminary experiments, similar adsorption behaviors were observed for all resins. Therefore, batch experiments were performed by using MAER-3 only, and the results were depicted in Fig. 6. As can be seen, the adsorption amounts of HA by MAER-3 decreased substantially with the concentrations of Ca^2+^ and Mg^2+^ increasing from 0 to 10.0 mmol L^−1^, which was 24.39% decrease for Ca^2+^ and 10.79% decrease for Mg^2+^, respectively. These results could be explained by the obvious decline in zeta potentials of HA solution. Although chelating interactions between alkaline-earth metal ions and HA molecules are weak, they can still form complexes and neutralize the negative charge of HA molecules, thus decrease the adsorptive force^28,38^. In addition, Ca^2+^ exerted a more pronounced influence on HA removal than Mg^2+^, which was mainly due to the lower affinity between Mg^2+^ and carboxylic groups of HA^29^. Interestingly, it was found that the HA adsorption capacity had a limited rise with the concentrations of cations increasing from 5 to 10 mmol L^−1^. It is reasonable to assume that the cations could also decrease the electrostatic repulsion between free and adsorbed HA macromolecules, and create new sites for adsorption. Besides, the coiling of cation-HA complexes can reduce the molecular size and solubility of HA (known as the salting-out effect), leading to an enhancement in HA adsorption capacities.

### Desorption and reusability

With the aim of industrial-scale applications, the reusability of MAERs is of great importance^20,39^. Due to the large molecular size and high hydrophobicity of HA, it could seriously hamper the adsorption and desorption behaviors by pore blocking or site shielding^40^. Brine is typically used for anion exchange resin regeneration as it can replace the adsorbed anionic adsorbate^41,42^. However, the hydrophobic interaction between adsorbents and adsorbate may be enhanced with the increasing NaCl in solution, which results in the fouling of resins.

In this study, desorption experiments were carried out by using 10% NaCl solution or a mixture of 10% NaCl + 1% NaOH solution as the desorption agent, respectively. As shown in Fig. S5, the desorption efficiencies (*DEs*) by NaCl/NaOH mixture were much higher than that of NaCl solution only, which was reasonable for the reduced hydrophobic interaction and dissociation of HA molecules in the presence of NaOH. Furthermore, the MAER-3 exhibited better desorption performance than other resins by using pure NaCl solution, which may be attributed to its obviously enhanced hydrophilicity and supermacroporous structure on the surface. This being the case, the improved anti-fouling performance of MAER-3 was directly on account of its higher DAI content in composition.

**Figure 7:**
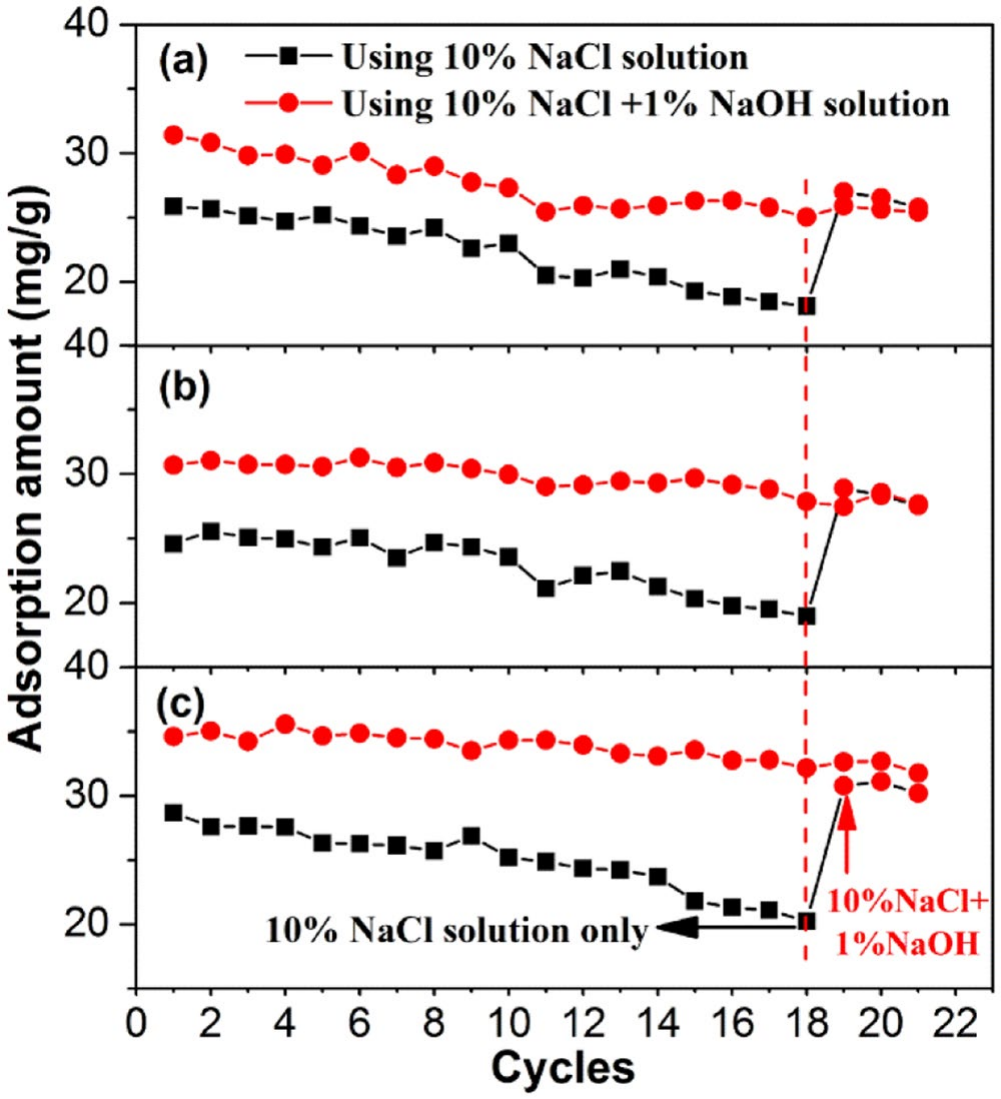
The adsorption amounts of HA during twenty-one adsorption cycles by (**a**) MAER-1, (**b**) MAER-2, and (**c**) MAER-3 (1.0 g resin/L, 50 mg/L of HA solution, 293 K).

**Figure 8:**
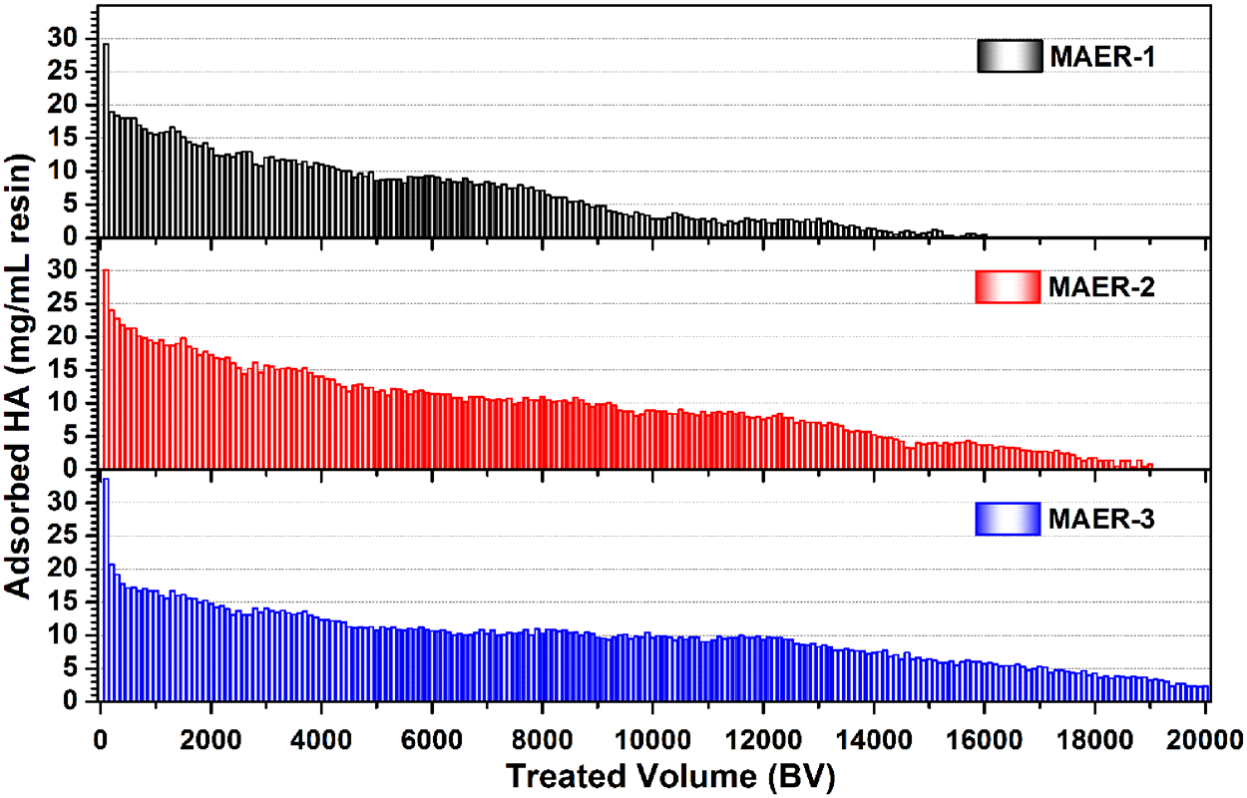
The HA adsorption performances by using MAERs in sequencing batch mode (1.0 mL resin, 50 mg/L of HA solution, 293 K).

The repeated adsorption-desorption processes were performed for 21 times to verify the reusability of MAERs. As shown in Fig. 7, the decline trends of HA adsorption for all resins were observed. After 18 adsorption-desorption cycles, the total decreases in HA adsorption capacity regenerated by NaCl/NaOH mixture were 20.34%, 9.26% and 7.09% for the recycling MAER-1, MAER-2, and MAER-3, respecitvely, while that by pure NaCl solution were 30.03%, 24.36%, and 26.83%, respectively. Moreover, the residual HA on resins due to the insufficient desorption by NaCl solution could be again desorbed by a mixture of NaCl/NaOH solution, as verified by the last three cycles. Also, the resins with larger DAI content, which meant higher hydrophilicity, exhibited better reusability potential, especially for MAER-3. Furthermore, as depicted in Fig. 8, an efficient adsorption of HA was observed for more than 15000 bed volume (BV) HA solution. The gradual loss of removal efficiency was attributed to the progressive loss of resin capacity during multiple cycles of HA removal. The saturated capacities of MAER-1, MAER-2 and MAER-3 for HA adsorption were 1118.9, 1714.6 and 1896.7 mg/mL resin, respectively, demonstrating their huge potential for HA adsorption. Based on the advantages of superior reusability, high adsorption capacity and convenient separability, the MAER-3 can be considered as an environmentally friendly and reusable adsorbent for NOM removal.

### Mechanism

SEM morphologies of single-bead and resin surface before and after adsorption of HA were observed in Fig. S6. No obvious differences were found for the overall morphologies of three resins. However, it was evident that adsorption of HA caused obviously morphological alteration on the exterior of resin surface. The thicker layer of surface coverage and masking of adsorptive sites by HA molecules suggested that the monolayer adsorption by electrostatic attraction may turn into multilayer adsorption by physical forces, which was formerly indicated by the Freundlich isotherm fitting.

The FTIR results also shed light on the nature of adsorptive interactions between HA and MAERs. As shown in Fig. S7a, slight changes were observed in the FTIR spectra of the two resins. However, the peaks at 1567.9 cm^−1^ for MAER-1 and 1571.7 cm^−1^ for MAER-3 were corresponding to the C=O of the resin matrix, which shifted to 1564.0 cm^−1^ and 1565.9 cm^−1^, respectively, indicating that the carbonyl groups may form hydrogen bonding with the un-ionized HA. Besides, the FTIR results at acidic conditions were shown in Fig. S7b. The peaks of C–O–C groups of the resin matrix were shifted from 1159 cm^−1^ to about 1147 cm^−1^, confirming the interactions between HA molecules and the oxygen atoms of C–O–C, and possible formation of oxonium groups (Fig. S8). The effects of oxygen atom of C–O–C for adsorption have been discussed in previous literatures^35,43–45^. It is said that both oxygen atoms of carbonyl and ester groups have a lone pair of electrons that can bind a proton ion through an electron pair sharing and form a complex. According to Yang *et al*.^35^, the existence of oxonium groups was confirmed during the adsorption of aromatic sulfonate by acrylic ester resin in an acidified solution.

**Figure 9:**
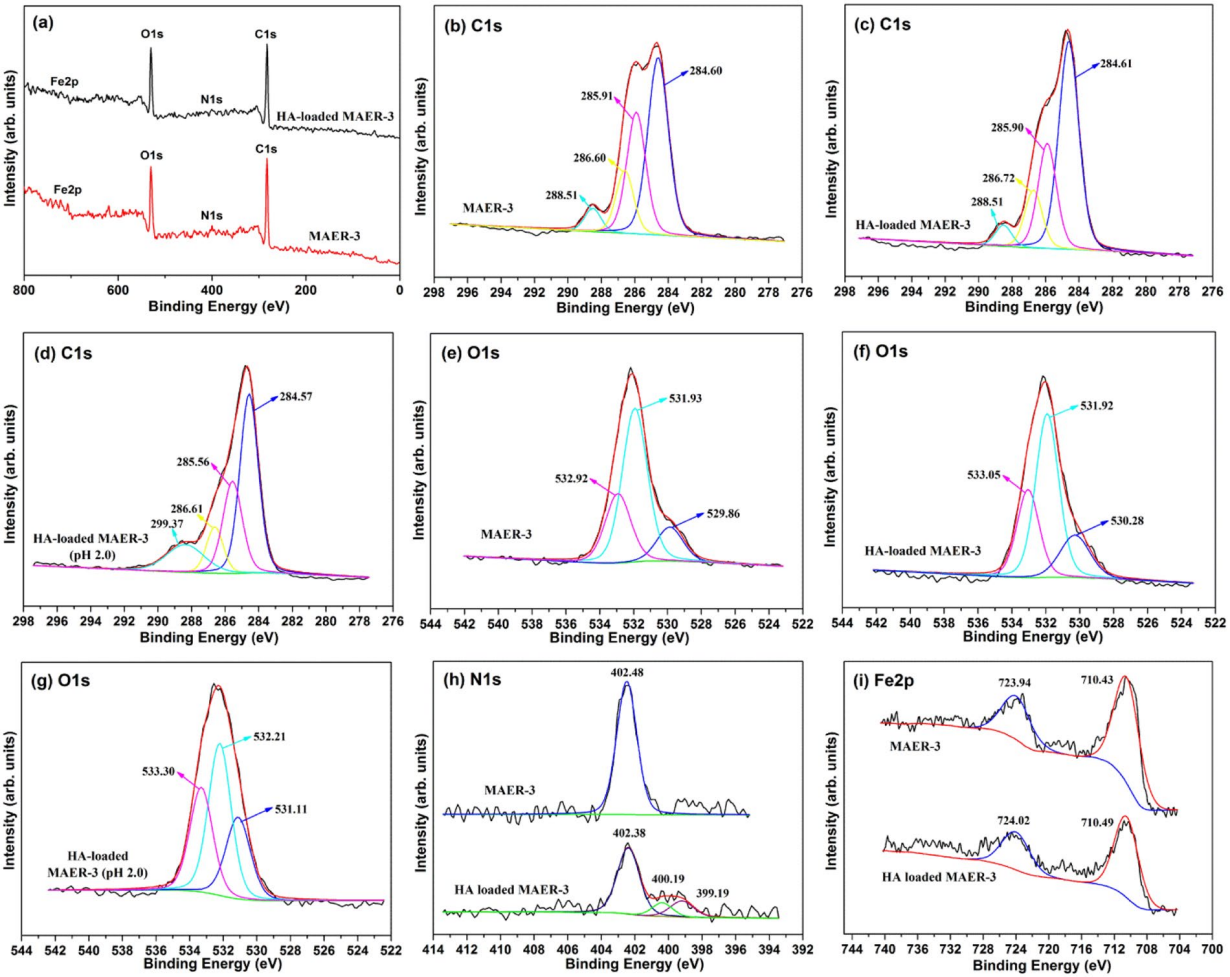
XPS spectra of (**a**) wide scan of virgin and HA-loaded MAER-3, (**b**–**d**) C1s spectra of virgin, HA-loaded and acidic HA-loaded MAER-3, (**e**–**g**) O1s spectra of virgin, HA-loaded and acidic HA-loaded MAER-3, (**h**) N1s spectra of virgin and HA-loaded MAER-3, and (**i**) Fe2p spectra of virgin and HA-loaded MAER-3.

XPS characterization was conducted to elucidate the adsorption mechanisms. The high-resolution C1s, O1s, N1s, Fe2p spectra as well as the wide scans of virgin and HA-saturated resin samples were shown in Fig. 9. The deconvolution of C1s spectra for MAER-3 was resolved into four peaks centered at 284.60, 285.91, 286.60, and 288.51 eV, which was attributed to the C–C/C–H, C–O–C/C–OH, C–N^+^, and O–C=O groups, respectively^35^. After HA uptake, the shifts of four peaks in C1s spectra were negligible. However, for the C1s spectra of HA-loaded MAER-3 at pH 2.0, the contents of O–C=O increased greatly, while the peak corresponding to carbon atoms of –CH2–O– at 285.91 eV shifted to 285.56 eV, indicating that this group played important roles in the HA adsorption. These results also suggested the possible formation of oxonium groups at the acidic condition.

The O1s spectra of MAER-3 were divided into three sub-peaks at 529.86, 531.93 and 532.92 eV, which were assigned to the C=O, O–H, and C–O, respectively^46^. After HA loading, the peaks of C=O and C–O shifted to 530.28 and 533.05 eV, while no obvious shift was observed for O–H groups. Additionally, the O1s sub-peaks for HA-loaded MAER-3 at pH 2.0 shifted greatly, along with the intensity of C=O and C–O peaks increased obviously. The N1s spectra of MAER-3 had a single peak at 402.48 eV for the quaternary ammonium-type N^29,32^. After HA adsorption, this peak shifted slightly to 402.38 eV, while two other peaks at lower binding energies appeared, which was definitely derived from the N-containing groups of HA molecules (N content of 3.73% for HA) like –CN, –N=, –NH–/–HH_2_, –OCONH–, and –CONH_2_^40^. In the Fe2p spectra, the broad peaks of Fe2p3/2 and Fe2p1/2 spectra were positioned at 710.43 and 723.94 eV, respectively. After HA adsorption, no obvious changes were found for Fe2p spectra, which also confirmed the non-interaction between HA molecules and γ-Fe_2_O_3_.

## Conclusions

In this study, a series of novel permanent-magnetic anion exchange resins called MAERs were developed and applied for HA removal. The MAERs were about 50–150 μm in size with uniform grain diameter and exhibited high rates of settlement in aqueous solution due to their superior magnetic characteristics. The use of DAI as the crosslinker can significantly improve the hydrophilicity, strength and adsorption capacity of resins, thereby suggesting a feasible method to enhance the adsorptive and anti-fouling performance of resins. The kinetic studies showed that the HA adsorption can be well fitted by pseudo-second-order and intra-particle diffusion model, and the diffusion constants increased with the increasing DAI content. Furthermore, the Freundlich isotherm model was more suitable for explaining the HA adsorption onto MAERs. The thermodynamic analysis of the adsorption process showed that the adsorption of HA by MAERs was an endothermic spontaneous reaction. The HA uptake depended highly on the solution pH and increased gradually as pH increased from 3.0 to 11.0. On the other hand, with the increase in the concentrations of co-existing cations (Ca^2+^ or Mg^2+^), the adsorption capacities decreased drastically at initial and increased a little at high cation concentrations. Ion exchange was proposed as the main adsorption mechanism, while hydrogen bonding, π-π interaction, cation-π bonding and formation of oxonium groups also played important roles in the HA adsorption. The HA-saturated MAERs can be efficiently regenerated by a mixture of NaCl/NaOH solution (10%/1%). During twenty-one adsorption-desorption cycles, the MAER-3 could be reused without an obvious decline in adsorption capacity, indicating a superior anti-fouling performance for HA removal. Taken together, the high capacity, fast kinetics, excellent reusability and convenient separability of MAER-3 made it a good candidate for NOM removal in water treatment.

## Electronic supplementary material

Supplementary information
