# Supplementary material for: Preparation of Permanent Magnetic Resin Crosslinking by Diallyl Itaconate and Its Adsorptive and Anti-fouling Behaviors for Humic Acid Removal
Source: Sci Rep. 2017 Dec 6;7:17103. doi: 10.1038/s41598-017-17360-8 (PMC5719021; doi:10.1038/s41598-017-17360-8)
Supplement: Supplementary file 1 — Supplementary information [file 41598_2017_17360_MOESM1_ESM.pdf]

Supplementary Information for

Preparation of Permanent Magnetic Resin Crosslinking by Diallyl Itaconate and Its Adsorptive and Anti-fouling Behaviors for Humic Acid Removal

Qimeng Li, Ji Wu, Ming Hua, Guang Zhang, Wentao Li, Chendong Shuang*, Aimin Li*

State Key Laboratory of Pollution Control and Resources Reuse, School of the Environment, Nanjing University, Nanjing 210023, P. R. China

*Corresponding authors.

Dr. Chendong Shuang

E-mail: shuangchendong@nju.edu.cn

Prof. Aimin Li

E-mail: liaimin@nju.edu.cn

Tel.: +86-25-89680377; fax: +86-25-89680377.

Contents

Text S1 Preparation of DAI

Text S2 Preparation of MAERs

Text S3 Adsorption kinetics model

Table S1 Sample code and feed compositions of resin samples

Table S2 Physicochemical properties of used adsorbents

Table S3 Thermodynamic parameters calculated at different adsorption amounts of HA by MAERs

Figure S1 The size distribution curves of MAERs microbeads

Figure S2 The adsorption capacity of HA onto three precursor resins and γ-Fe_2O_3 (1.0 g resin/L, 100 mg/L of HA solution, 293K)

Figure S3 The adsorption capacity of HA onto different adsorbents (1.0 g adsorbent/L, 100 mg/L of HA solution, 293 K)

Figure S4 The zeta potentials (a) and size distribution (b) of HA solution as a function of solution pH

Figure S5 Desorption kinetics of saturated resins at 293 K using (a) 10% NaCl solution and (b) a mixture of 10% NaCl + 1% NaOH solution

Figure S6 SEM micrographs of single-bead and surface morphology of virgin and saturated resins: A, B, and C represent the virgin resin, while a, b, and c represent the saturated resin

Figure S7 FTIR spectra of MAER-1 and MAER-3 at (a) normal condition, (b) acidic condition (pH 2.0 and 3.0)

Figure S8 The adsorption of HA on MAERs by ① hydrogen bonding and ② electrostatic attraction of positively charged oxonium group at strongly acidic conditions (R and R' represent matrix of MAERs)

Text S1 Preparation of DAI

Diallyl itaconate (DAI), as a hydrophilic crosslinker, could be used for preparation of polyacrylic resin. The DAI was obtained by the esterification reaction of propanol and itaconic acid with a small amount of 70 % H_2SO_4 as the catalyst. The propanol and itaconic acid were purchased from BST Tianjin Co., Ltd and Hangzhou Yunuo Chemical Co., Ltd respectively. Although the mole ratio in the equation for propanol and itaconic acid was 2:1, in fact, the actual stoichiometric ratio of propanol and itaconic acid was 3:1 for proceeding completely and decreasing the reaction time. The temperature was maintained in the range of 376 ~ 396 K. At pre-determined time intervals (3 h, 6 h, 9 h, 12 h ···), 1 mL of the solution was withdrawn and measured. Upon completion, the DAI was purified by vacuum distillation.

Text S2 Preparation of MAERs

The precursor resins were prepared by suspension polymerization of GMA (85.0 g) and desired amount of crosslinker DVB or/and DAI in a 1 L three-necked flask. The modified γ-Fe_2O_3 (60.0 g) particles were also stirred with monomers. BPO (1.5 g) and cyclohexanol (70.0 g) were used as the initiator and porogen, respectively. Next, the water dispersion phase containing 500 mL water, 0.50 g GH20 and 0.50 g Na_2SO_4 was transferred into the flask and stirred. The mixture was gradually elevated to 75 ^oC and maintained for 12 h. After that, the resultant precursor resins (100.0 g) were aminated with trimethylamine hydrochloride solution (350 mL, 50% w/w) at 70 ^oC for 10 h with continuous stirring. Prior to use, the resins were purified with methanol and acetone for 12 h by Soxhlet extraction. Upon completion, the obtained resins were repeatedly rinsed to neutral with deionized water and dried to constant weight.

Text S3 Adsorption kinetics models

To elucidate the adsorption behaviors of three resins, the pseudo-first order, pseudo-second order and Weber-Morris intra-particle diffusion models were applied and expressed as follows:

Pseudo-first-order kinetic model: $Q_t = Q_e(1 - \exp(-k_1 t))$ (1)

Pseudo-second-order kinetic model: $Q_t = (k_2 t Q_e^2)/(1 + k_2 t Q_e)$ (2)

$$h = k_2 Q_e^{\,2} \quad (3)$$

Intra-particle diffusion model: $Q_t = K_{id} t^{0.5} + I$ (4)

Where, Q_e (mg g^{-1}) and Q_t (mg g^{-1}) refer to the amounts of adsorbed HA at equilibrium and time t, respectively. The k_1 (min^{-1}) and k_2 (g (mg min)$^{-1}$) are the constants of pseudo-first-order and pseudo-second-order model, respectively. The h (mg (g min)$^{-1}$) is the initial adsorption rate. K_{id} (mg (g min)$^{0.5}$) is the constant of intra-particle diffusion model.

Table S1

Table S1 Sample code and feed compositions of resin samples[a]

Sample code	GMA (g)	DVB (g)	DAI (g)	Cyclohexanol (g)
MAER-1	85	15	-	70
MAER-2	85	7.5	7.5	70
MAER-3	85	-	15	70

[a] For all resins, the dosage of BPO and modified γ-Fe_2O_3 were 1.5 g and 60.0 g, respectively.

Table S2

Table S2 Physicochemical properties of used adsorbents

Resin	D213	MAER-3	D201	XAD-4	XAD-8	F400D
Skeleton	Polyacrylic-divinylbenzene	Polyacrylic-divinylbenzene	Styrene-divinylbenzene	Styrene-divinylbenzene	Polyacrylic-divinylbenzene	Carbon
Functional groups	$-N^+(CH_3)_3\ Cl^-$	$-N^+(CH_3)_3\ Cl^-$	$-N^+(CH_3)_3\ Cl^-$	-	-	-
Exchange capacity (mmol/g)	3.90	3.14	4.10	-	-	-
Water content (%)	58.50	68.53	56.70	50.10	32.10	37.10
Diameter (mm)	0.3 ~ 0.8	0.05 ~ 0.15	0.4 ~ 0.7	0.4 ~ 0.7	0.4 ~ 0.7	0.8-1.2
Specific surface area (m^2/g)	13.63	1.84	3.14	950.91	169.32	1130.72
Average pore diameter (nm)	5.87	16.85	4.63	5.24	9.37	2.26

Table S3

Table S3 Thermodynamic parameters calculated at different adsorption amounts of HA by MAERs

Resin	Given Q_e (mg/g)	ΔH (kJ/mol)	ΔG (kJ/mol)			ΔS (J/mol K)		
			278 K	293 K	308 K	278 K	293 K	308 K
MAER-1	75	41.17	-3.448	-4.063	-5.178	160.50	154.38	150.48
	100	37.59				147.62	142.16	138.86
	125	34.78				137.51	132.57	129.73
MAER-2	75	48.64	-3.243	-3.951	-5.966	186.63	179.49	177.29
	100	42.34				163.97	157.99	156.84
	125	37.48				146.49	141.40	141.06
MAER-3	75	43.86	-3.754	-4.585	-5.767	171.27	165.34	161.13
	100	39.80				156.67	151.48	147.94
	125	36.63				145.27	140.67	137.65

Figure S1

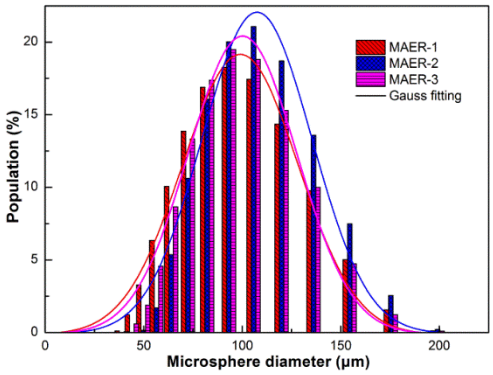

Fig. S1 The size distribution curves of MAERs microbeads

Figure S2

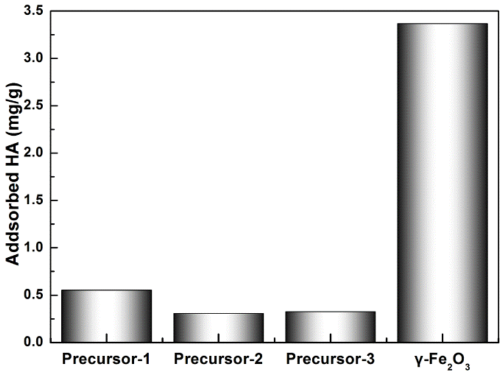

Fig. S2 The adsorption capacity of HA onto three precursor resins and γ-Fe_2O_3 (1.0 g resin/L, 100 mg/L of HA solution, 293K)

Figure S3

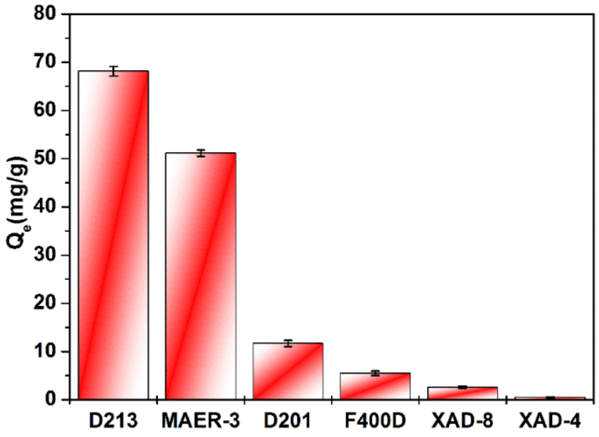

Fig. S3 The adsorption capacity of HA onto different adsorbents (1.0 g adsorbent/L, 100 mg/L of HA solution, 293 K)

Figure S4

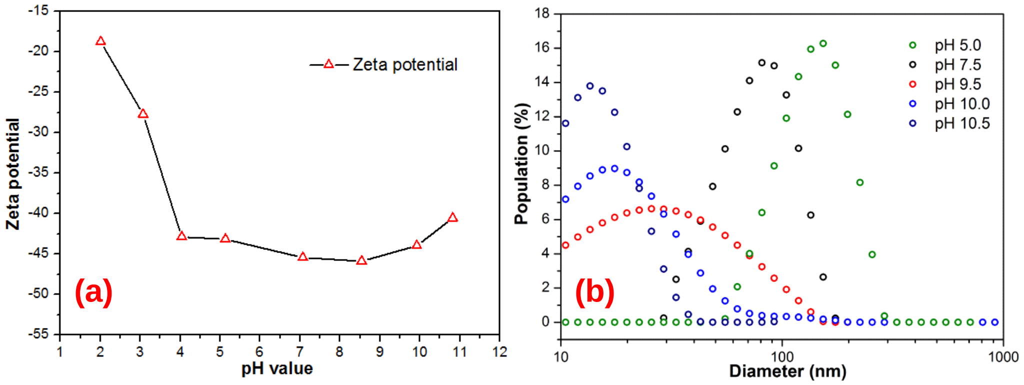

Fig. S4 The zeta potentials (a) and size distribution (b) of HA solution as a function of solution pH

Figure S5

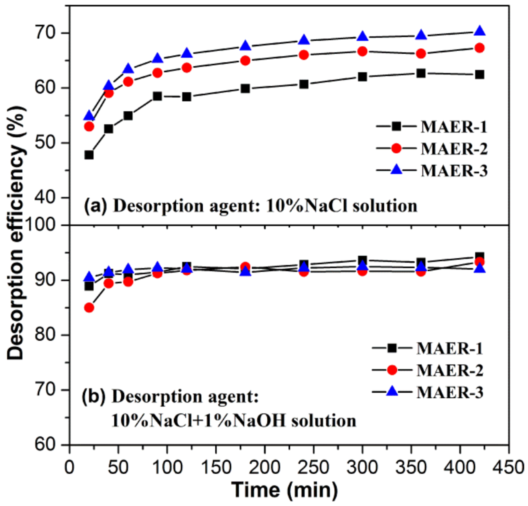

Fig. S5 Desorption kinetics of saturated resins at 293 K using (a) 10% NaCl solution and (b) a mixture of 10% NaCl + 1% NaOH solution

Figure S6

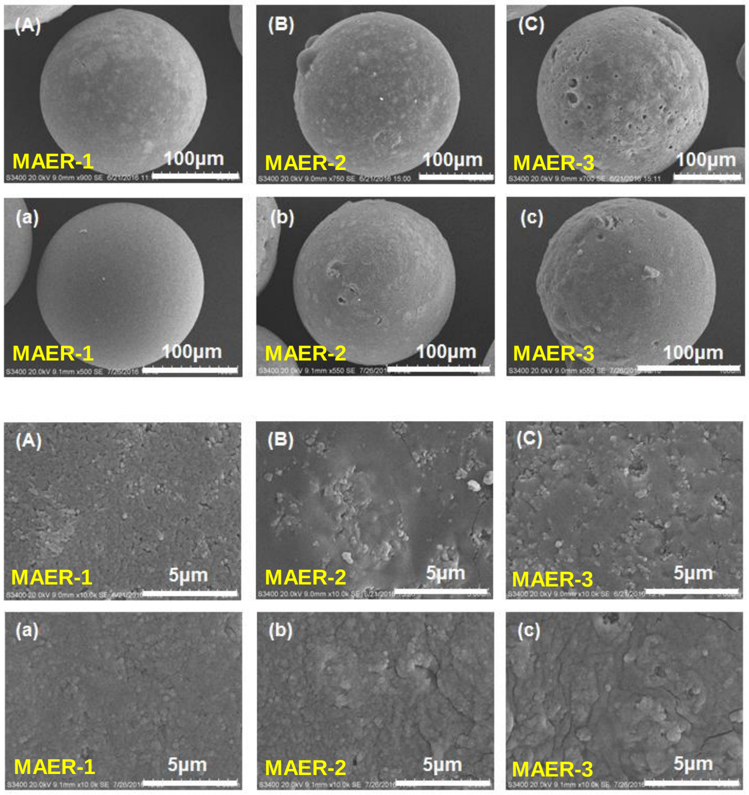

Fig. S6 SEM micrographs of single-bead and surface morphology of virgin and saturated resins: A, B, and C represent the virgin resin, while a, b, and c represent the saturated resin

Figure S7

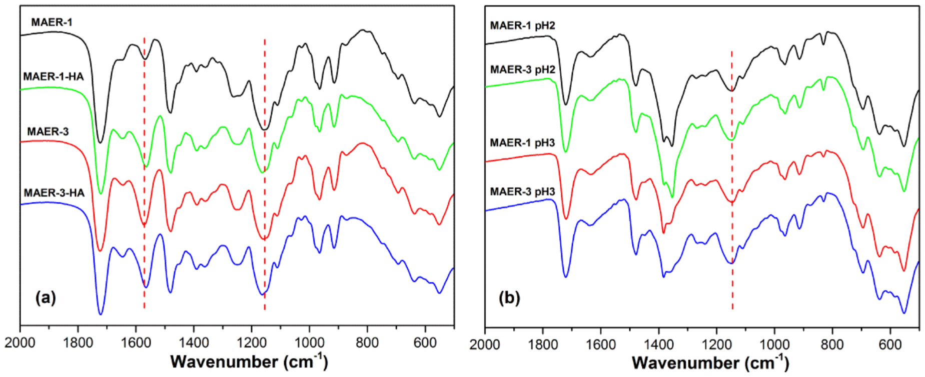

Fig. S7 FTIR spectra of MAER-1 and MAER-3 at (a) normal condition, (b) acidic condition (pH 2.0 and 3.0)

Figure S8

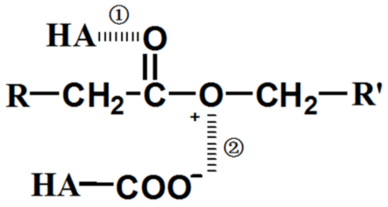

Fig.S8 The adsorption of HA on MAERs by ① hydrogen bonding and ② electrostatic attraction of positively charged oxonium group at strongly acidic conditions (R and R' represent matrix of MAERs)
